# Acute pancreatitis following digital subclavian artery angiography: a case report and literature review

**DOI:** 10.1093/jscr/rjag126

**Published:** 2026-03-11

**Authors:** Xinjuan Xu, Yixin Sun, Yong He, Lei Xi, Ruilin Ren, Ning Ma, Yufeng Wang

**Affiliations:** Department of Neurosurgery, The Affiliated Cardiovascular Hospital of Shanxi Medical University and Shanxi Cardiovascular Hospital, No. 18, Yifen street, Wanbailin District, Taiyuan City, Shanxi Province, 030024, China; Department of Hepatobiliary and Pancreatic Surgery, Peking University First Hospital, No. 8, Xishiku Street, Xicheng District, Beijing, 100034, China; Department of Neurosurgery, The Affiliated Cardiovascular Hospital of Shanxi Medical University and Shanxi Cardiovascular Hospital, No. 18, Yifen street, Wanbailin District, Taiyuan City, Shanxi Province, 030024, China; Department of Neurosurgery, The Affiliated Cardiovascular Hospital of Shanxi Medical University and Shanxi Cardiovascular Hospital, No. 18, Yifen street, Wanbailin District, Taiyuan City, Shanxi Province, 030024, China; Department of Neurosurgery, The Affiliated Cardiovascular Hospital of Shanxi Medical University and Shanxi Cardiovascular Hospital, No. 18, Yifen street, Wanbailin District, Taiyuan City, Shanxi Province, 030024, China; Department of Neurosurgery, The Affiliated Cardiovascular Hospital of Shanxi Medical University and Shanxi Cardiovascular Hospital, No. 18, Yifen street, Wanbailin District, Taiyuan City, Shanxi Province, 030024, China; Department of Neurosurgery, The Affiliated Cardiovascular Hospital of Shanxi Medical University and Shanxi Cardiovascular Hospital, No. 18, Yifen street, Wanbailin District, Taiyuan City, Shanxi Province, 030024, China

**Keywords:** contrast agent toxicity, acute pancreatitis, subclavian artery angiography

## Abstract

Subclavian artery (SA) angiography has many well-documented complications. Acute pancreatitis (AP) is a rarely described complication with potentially life-threatening repercussions. This article reports the case of a man with AP that occurred the second day after SA angiography. Contrast agent toxicity and cholesterol emboli are the two mechanisms involved in the occurrence of AP after SA angiography. We searched previous literature using PubMed databases during the same period as comparison.

## Introduction

Left subclavian artery (SA) occlusion with steal syndrome is a common cause of posterior circulation ischemia, characterized by recurrent dizziness, unsteady gait, and visual disturbances—symptoms driven by retrograde blood flow from the vertebral artery to the occluded SA [1]. Vascular revascularization are first-line treatment [2], but their complications typically include access-site issues (e.g. hematoma, pseudoaneurysm) or renal dysfunction, with acute pancreatitis (AP) being extremely rare [3].

To date, only 10 cases of AP following coronary angiography have been reported in the literature, with contrast agent toxicity and cholesterol emboli identified as the two key pathophysiological mechanisms. This case is clinically valuable because it describes AP after SA recanalization—an underreported scenario—and provides insights into differential diagnosis and management of post-interventional abdominal symptoms.

## Case presentation

On 13 May 2025, a 63-year-old male experienced sudden-onset dizziness, headache, unsteady gait, and blurred vision; these symptoms resolved spontaneously within 30 minutes. Similar symptoms recurred on 23 May, lasting 10 minutes, with a subsequent episode of dizziness and unsteady gait 30 minutes later while climbing a slope. Digital subtraction angiography performed on 27 May revealed left SA occlusion consistent with subclavian steal syndrome. On 1 June, he experienced another episode of dizziness and blurred vision lasting one hour, after which there was no further recurrence.

The patient had a medical history of hypertension and cervical spondylosis surgery, a 40-year smoking history, and no history of alcohol use. Long-term medications included indobufen and rosuvastatin calcium. Laboratory investigations showed normal blood counts, glucose, liver, and renal function, but marked hyperhomocysteinemia (39.7 μmol/l) (Table 1). He was subsequently transferred to our hospital for further management.

**Table 1:** Laboratory data on admission

**Indicator**	**Reference Range**	**On admission**
WBC count	4.0–10.0 × 10^9^/l	9.74 × 10^9^/l
Lymphocyte count	1.1–3.2 × 10^9^/l	3.57 × 10^9^/l
ALT	7–40 U/l	146.4 U/l
Total cholesterol	2.83–5.2 mmol/l	3.42 mmol/l
Triglycerides	0.56–1.7 mmol/l	2.38 mmol/l
Low-density lipoprotein cholesterol (LDL-C)	<3.4 mmol/l	2.32 mmol/l
Glucose	3.9–6.1 mmol/l	4.7 mmol/l
Creatinine	53–106 μmol/l	112.5 μmol/l
Urea	2.9–8.2 mmol/l	9.5 mmol/l
Homocysteine	5.0–15.0 μmol/l	39.7 μmol/l

On 3 June 2025, the patient underwent left SA occlusion recanalization via the right transradial approach. Given the complexity of navigating through the SA total occlusion and the imperative to repeatedly confirm true lumen position and assess collateral circulation, the procedure required extensive fluoroscopic guidance with multiple contrast injections. Consequently, 600 ml of iodixanol contrast medium (Visipaque, 320 mgI/ml, non-ionic low-osmolar) was administered intraoperatively. The complex procedure lasted ~3 hours, with no immediate complications.

On postoperative day 1, the patients’ abdominal examination was soft and flat, without tenderness or rebound tenderness. By postoperative day 2, localized tenderness developed in the right lower quadrant (pain score 4/10 on the Numeric Rating Scale). Serum amylase was elevated to 288 U/l. There was no rebound tenderness, jaundice, ascites, or palpable masses.

**Figure 1:**
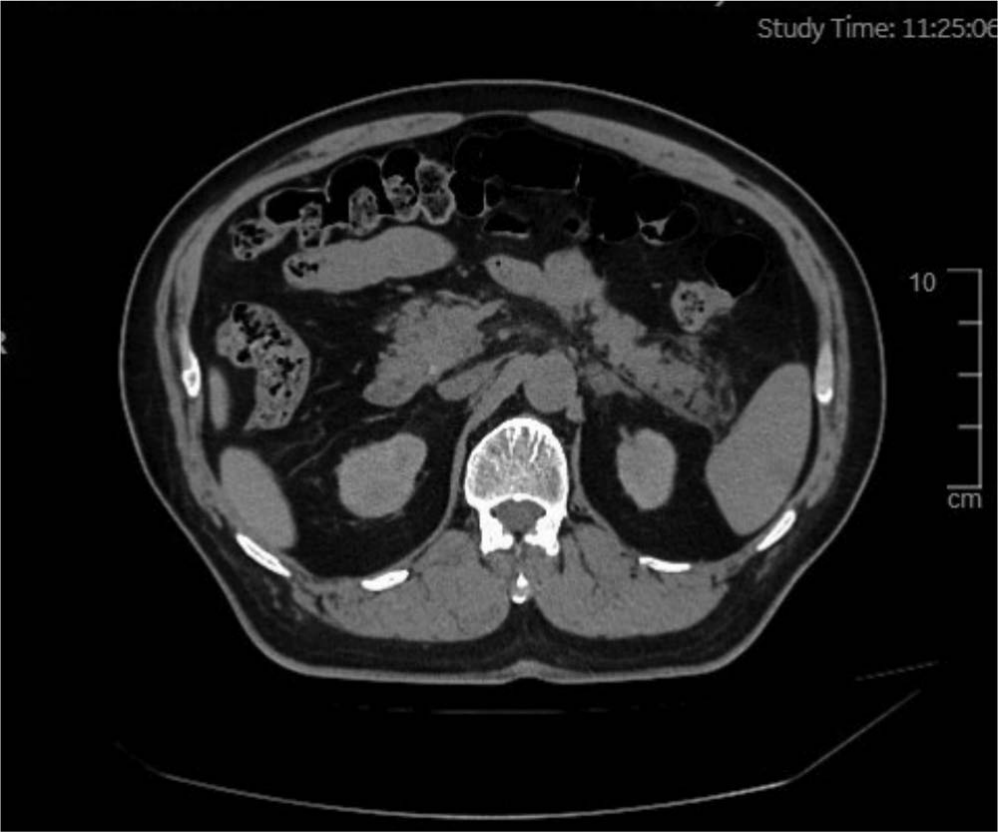
Abdominal scan showing an area of exudative changes within the tail of the pancreas (Balthazar grade B).

Diagnostic workup included a normal electrocardiogram (sinus rhythm, 68 beats/min), which ruled out a cardiac etiology for the abdominal pain. Abdominal Ultrasound (5 June 2025) revealed findings of fatty liver and multiple hepatic cysts (largest 1.5 cm), postprandial gallbladder (no stones/wall thickening), and unremarkable spleen (noting ultrasound limitations for early pancreatitis). However, ultrasound has inherent limitations for early pancreatitis. Given the clinical suspicion for pancreatitis and the need for cross-sectional imaging while prioritizing patient safety due to the substantial intraoperative contrast load already administered, an abdominal non-contrast computed tomography (NCCT) was purposefully obtained on 5 June 2025. This study confirmed the hepatic cysts and additionally identified bilateral renal cysts (largest 1.2 cm). Crucially, it demonstrated findings consistent with mild-to-moderate AP (Balthazar Grade B), characterized by pancreatic tail exudation without evidence of necrosis or peripancreatic fluid collection (Fig. 1).

For post-interventional AP, supportive treatment was initiated per contrast-induced pancreatitis pathophysiology and World Health Organization AP guidelines to maintain urine output >0.5 ml/kg/h, correcting hypovolemia from pancreatic inflammation and contrast-related hyperviscosity. Octreotide acetate (0.1 mg subcutaneous q8h) inhibited pancreatic exocrine function; intravenous pantoprazole (40 mg daily) reduced acid-mediated pancreatic stimulation; and intramuscular hyoscine butylbromide (20 mg prn) relieved abdominal spasms. The patient maintained NPO status for 5 days to rest the pancreas (due to persistent abdominal pain/nausea), transitioning to a low-fat diet (<30 g/day) on postoperative day 6.

Abdominal pain resolved, and amylase decreased to 104 U/l (near normal) on postoperative day 5; no discomfort (dizziness, abdominal pain, visual disturbances) was reported until discharge. Laboratory results (10 June 2025) showed elevated white blood cell (WBC) (15.66 × 10^9^/l), neutrophil percentage (79.4%), monocyte count (1.04 × 10^9^/l), and CRP (65.25 mg/l, trending downward with resolving inflammation), alongside normal amylase (104 U/l), alanine aminotransferase (ALT) (8.1 U/l), creatinine (102 μmol/l), and urea (8.6 μmol/l).

A literature review (1981–2020) of post-interventional pancreatitis identified 8 studies involving 56 patients, including cardiac catheterization (*n* = 3), coronary angiography (with/without stenting, *n* = 3), and arteriovenous (AV) dialysis access thrombectomy (*n* = 2) (Table 2). Of these, six studies (*n* = 41) explicitly correlated pancreatitis with intravascular contrast media, supported by etiological evidence of contrast-induced toxicity or hemodynamic effects.

**Table 2:** Summary of previous studies

**First author of literature**	**Procedure causing pancreatitis**	**Pancreatic events occurred**	**Number of cases**	**Prognosis**	**Suspected etiology**
Orvar 1994 [4]	Cardiac catheterization and aortic angiography	AP, some complicated with extensive pancreatic necrosis and pseudocyst formation	Nine cases (screened from 44 episodes)	Three cases succumbed to pancreatitis-related complications; six cases exhibited self-limiting clinical manifestations (Ranson score: 1–2)	Atherosclerotic cholesterol embolism syndrome (autopsy-confirmed with multi-organ involvement)
Drost 1984 [5]	Left heart catheterization (femoral arterial approach)	No specific pancreatic events were clearly documented (cholesterol embolism with multi-organ involvement)	Seven patients diagnosed with cholesterol embolism	Four cases died within 4.5 months (not directly attributed to embolism); three cases required toe amputation due to peripheral ischemia	Cholesterol embolism (autopsy-verified embolization in kidneys and other visceral organs)
Krauer 2020 [6]	Coronary angiography with stent implantation	AP (Balthazar grade C), accompanied by pancreatic head necrosis and necrotic effusion	One case	Clinical improvement achieved after 3 weeks of hospitalization; no complications observed during re-angiography at 6-month follow-up	Contrast medium-induced toxicity (iodixanol, 225 ml); cholesterol embolism (not confirmed)
Hajimaghsoudi 2017 [7]	Coronary angiography	Acute necrotizing pancreatitis, with pancreatic head enlargement and perihepatic effusion	One case	Discharged after clinical improvement following 5 days of conservative management	Contrast medium-induced toxicity (iodixanol, 100 ml); cholesterol embolism (excluded by diagnostic workup)
Gorges 2013 [8]	Cardiac catheterization (coronary angiography combined with ventricular angiography)	AP, secondary to acute respiratory distress syndrome (ARDS)	One case	Clinical resolution achieved within 48 hours; serum amylase and lipase levels normalized at discharge	Contrast medium-induced toxicity (iopamidol, 120 ml); cholesterol embolism (ruled out)
Chin and Ng 1981 [9]	Transfemoral left ventricular angiography	Acute fulminant hemorrhagic pancreatitis	One case	Expired 48 hours after disease onset	Pancreatic parenchymal injury induced by intra-arterial infusion of contrast medium (diatrizoate meglumine, 40 ml)
Kheda *et al.* 2008 [10]	Intravenous administration of iodixanol-320 during AV dialysis access thrombectomy in patients with end-stage renal disease (ESRD)	Acute necrotizing pancreatitis; marked elevation of serum amylase (up to 5332 U/l) and serum lipase (up to 6423 U/l); CT suggestive of pancreatic necrosis	Two cases (both ESRD patients)	No recurrence of pancreatitis after switching to iohexol; symptom resolution and normalization of serum amylase/lipase following intravenous fluid resuscitation and empirical anti-infective therapy	Hyperviscosity of iodixanol (11.8 cP at room temperature) → increased plasma viscosity → reduced pancreatic blood flow → ischemic necrosis; underlying pancreatic pathology in ESRD patients may enhance susceptibility
Kheda *et al.* 2010 [11]	Randomized administration of iodixanol or iohexol during AV access thrombectomy/angioplasty in hemodialysis patients (without predisposing factors for pancreatitis)	Iodixanol group: 2 cases of AP, characterized by >2-fold elevation of serum amylase (up to 827 U/l) and serum lipase (up to 1460 U/l), accompanied by nausea, vomiting, and abdominal pain; Iohexol group: no pancreatitis cases	30 cases (15 in the iodixanol group, 15 in the iohexol group)	Symptom resolution within 3 days without hospitalization in the 2 affected cases of the iodixanol group; no severe complications or mortality in either group	Hyperviscosity of iodixanol (11.8 cP at 37°C, significantly higher than 6.3 cP of iohexol) → impaired pancreatic microcirculatory perfusion → ischemia; Iohexol maintains pancreatic blood flow via intravascular volume expansion due to its hyperosmolality (672 mOsm/kg H₂O), avoiding ischemic injury

Iodixanol was the most frequently implicated agent (four studies, *n* = 22 patients), with an AP incidence of 27.3% (six cases). Clinical manifestations included necrotizing pancreatitis (*n* = 3), Balthazar grade C pancreatitis (*n* = 1), and symptomatic hyperamylasemia/hyperlipasemia (*n* = 2). Pathogenic mechanisms included: (i) hyperviscosity (11.8 cP at 37°C), elevating plasma viscosity and impairing pancreatic perfusion (validated vs. iohexol in Kheda *et al.*, 2010) [11]; and (ii) synergistic effects with End Stage Renal Disease (ESRD)-related intrinsic pancreatic vulnerability [10]. Notably, switching to iohexol resolved recurrence in ESRD patients.

Iopamidol (120 ml) [8] and diatrizoate meglumine (40 ml) [9] each caused one case of AP. The iopamidol-associated case, complicated by acute respiratory distress syndrome, resolved within 48 hours, while diatrizoate meglumine induced fulminant hemorrhagic pancreatitis leading to death—indicating potential dose- or agent-specific toxicity (ionic diatrizoate meglumine may exhibit higher cytotoxicity than non-ionic iodixanol/iopamidol).

Notably, no pancreatitis events occurred in 15 hemodialysis patients undergoing AV access procedures with iohexol [11]. Its favorable safety profile was attributed to lower viscosity (6.3 cP at 37°C) and hyperosmolality-mediated intravascular volume expansion, which preserved pancreatic microcirculation—directly contrasting with iodixanol’s ischemic effects.

## Discussion

We report a 63-year-old male who developed AP shortly after endovascular revascularization for left SA occlusion, following intraprocedural administration of 600 ml iodixanol (Visipaque). The diagnosis was confirmed by postoperative abdominal pain, elevated serum amylase (288 U/l), and NCCT evidence of pancreatic tail exudation—consistent with contrast-induced AP. This case holds clinical significance by highlighting AP risk with high-dose iodixanol, emphasizing the roles of contrast viscosity and cumulative dose in pathogenesis, and illustrating the value of timely recognition and supportive management for favorable outcomes. Key clinical and mechanistic insights are discussed below, integrating this case with existing literature.

AP is a rare yet well-recognized iatrogenic complication of contrast-enhanced procedures, with an estimated incidence of <0.1% in the general population [12]. However, risk is amplified by specific patient factors (e.g. end-stage renal disease, preexisting pancreatic dysfunction) and procedural variables (e.g. contrast type, cumulative dose) [13]. This case is notable for two critical features: the high iodixanol dose (600 ml) and the patient’s underlying vascular risk profile, which may have exerted synergistic effects to exacerbate pancreatic injury.

In conventional clinical practice, iodinated contrast medium doses for peripheral vascular interventions are restricted to 100–200 ml, since doses >300 ml correlate with a dose-dependent increase in adverse events (e.g. nephrotoxicity, pancreatitis). The 600 ml iodixanol dose administered here—three times the standard threshold—likely exhausted the pancreatic microcirculatory reserve. Notably, iodixanol (an iso-osmolar nonionic dimer) has a viscosity of 11.8 cP at 37°C, far exceeding human plasma (1.72 cP) and monomeric contrast agents (e.g. iohexol, 3.3 cP) [14]. As evidenced by Kheda *et al.*’s pilot study and case reports [11], iodixanol-induced hyperviscosity is a key driver of AP: it elevates plasma viscosity, reduces capillary blood flow velocity, and triggers pancreatic ischemia, thereby inducing acinar cell necrosis and inflammatory cascade activation.

The patient’s baseline risk factors further underscore clinical relevance. Chronic hypertension (right upper extremity systolic BP up to 180 mmHg), 40-pack-year smoking history, and marked hyperhomocysteinemia (39.7 μmol/l; normal <15 μmol/l) are well-established vascular risk factors impairing endothelial function and microcirculatory integrity. Preclinical studies indicate that endothelial dysfunction exacerbates contrast-induced tissue hypoperfusion, as reduced nitric oxide bioavailability blunts vasodilatory capacity in response to viscosity-mediated flow reduction [10, 11]. While the patient did not have ESRD (creatinine 112.5 μmol/l at admission), his vascular comorbidities likely rendered the pancreatic microvasculature more susceptible to iodixanol’s hemodynamic effects—highlighting that AP risk is not limited to ESRD patients but extends to those with broader vascular dysfunction.

The pathogenesis of AP involves a multifactorial interplay between contrast medium properties and pancreatic physiology, with three core mechanisms supported by this case and existing literature: (i) iodixanol’s high viscosity disrupts the pancreas’s low-resistance microcirculatory bed. The pancreas relies on continuous capillary perfusion to maintain exocrine and endocrine function, and even mild reductions in flow can induce ischemia–reperfusion injury. As shown in Schmidt *et al.*’s animal model of necrotizing pancreatitis, contrast medium-induced reduction in low-flow capillary perfusion (by 30%–40%) and capillary stasis (15.9% vs. 3.2% in saline controls) directly contributes to acinar cell injury [15]. In this case, the high dose of iodixanol likely exacerbated viscosity-related flow reduction, particularly in the pancreatic tail—a region with relatively sparse collateral circulation, consistent with the CT finding of isolated tail exudation; (ii) beyond hemodynamic effects, iodixanol may exert direct toxic effects on pancreatic acinar cells. Jin *et al.* demonstrated that iodinated contrast media induce aberrant cytosolic Ca^2+^ signaling in acinar cells, activating calcineurin and nuclear factor-κB (NF-κB) pathways—key mediators of pancreatic inflammation and necrosis [16]. While this case did not include histopathological confirmation, the elevation in C-reactive protein (from 8.19 to 65.25 mg/l) and leukocytosis (16.3 × 10^9^/l) reflects a systemic inflammatory response consistent with NF-κB activation; (iii) the pancreas metabolizes and clears contrast medium via biliary and vascular routes. High cumulative doses may overwhelm these clearance mechanisms, leading to contrast medium accumulation in pancreatic ducts and interstitium. This accumulation exacerbates osmotic stress and ductal obstruction, further amplifying acinar cell injury. The 600 ml dose used here likely exceeded the pancreas’s clearance capacity, as even healthy individuals have a maximum contrast clearance rate of ~10 ml/min for iodinated agents.

To establish AP as the etiology, we rigorously excluded alternative causes of postoperative AP: Abdominal ultrasound and NCCT showed no cholelithiasis, choledocholithiasis, or biliary dilatation, ruling out biliary pancreatitis—the most common cause of AP in adults [13]. The patient’s long-term medications (indobufen, rosuvastatin) have minimal association with AP. Rosuvastatin-induced AP is reported in <0.01% of users, typically with prolonged use (>1 year) or high doses, and indobufen has no documented cases of AP in major pharmacovigilance databases. Serum triglycerides (2.38 mmol/l) were within the normal range, excluding hypertriglyceridemic pancreatitis (diagnostic threshold >11.3 mmol/l) [13]. Endovascular revascularization via radial access is minimally invasive and does not involve direct pancreatic manipulation. Postoperative stress-induced AP is rare and typically mild, with amylase elevations <2 upper limit of normal—unlike this case (amylase 288 U/l, ~3 × upper limit). The patient denied alcohol consumption, and serum transaminases (ALT 14.2 U/l) were normal, ruling out alcoholic or viral hepatitis-related pancreatitis. The temporal relationship (symptoms onset 24 hours post-contrast administration), dose-dependent risk, and exclusion of alternative etiologies strongly support AP as the diagnosis.

This case offers three key clinical takeaways for interventional cardiologists and radiologists:

Iodixanol’s hyperviscosity makes it a higher-risk agent for AP compared to low-viscosity alternatives (e.g. iohexol, iopamidol) [11]. When high doses are anticipated (e.g. complex vascular interventions), consideration of low-viscosity contrast media may reduce pancreatic injury risk. Additionally, dose should be minimized to the smallest volume necessary for adequate imaging—ideally <300 ml for peripheral vascular procedures.

Patients with vascular comorbidities (hypertension, smoking, hyperhomocysteinemia), preexisting pancreatic dysfunction, or prior contrast medium adverse events should be stratified as high-risk for AP. Postoperatively, monitoring of serum amylase/lipase (at 24–48 hours) and clinical assessment for abdominal pain, nausea, or vomiting are critical for early detection.

The patient’s favorable outcome—resolution of symptoms and normalization of amylase with supportive care (intravenous hydration, fasting, antiemetics) and octreotide—aligns with current AP management guidelines. Octreotide, a somatostatin analog, reduces pancreatic exocrine secretion and may mitigate acinar cell injury, though its role in AP is supported by observational data rather than randomized trials [13].

This case has limitations inherent to single-case reports: the lack of histopathological confirmation of pancreatic injury and the inability to definitively rule out unmeasured confounding factors (e.g. subclinical pancreatic steatosis). Future research should focus on prospective studies evaluating contrast medium viscosity, dose, and patient-specific risk factors in AP development, as well as randomized trials of prophylactic strategies (e.g. low-dose octreotide, preprocedural hydration) in high-risk populations.

In conclusion, this case highlights that high-dose iodixanol can induce AP in patients with underlying revascularization, likely via hyperviscosity-mediated microcirculatory impairment and direct cytotoxicity. Therefore, clinicians should exercise caution when using high doses of hyperviscous contrast media and implement targeted monitoring strategies in high-risk patients.

## Data Availability

No additional data are available.
